# The preprophase band is dispensable for robust cell division orientation in wood‐forming cambium stem cells

**DOI:** 10.1111/nph.71449

**Published:** 2026-07-20

**Authors:** Xiaomin Liu, Pantelis Livanos, Laura Sophie Schütz, Sabine Müller, Thomas Greb

**Affiliations:** ^1^ Heidelberg University, Centre for Organismal Studies (COS) 69120 Heidelberg Germany; ^2^ Department of Biology Friedrich‐Alexander University Erlangen‐Nuremberg 91058 Erlangen Germany; ^3^ Department of Biotechnology Agricultural University of Athens 11855 Athens Greece; ^4^ Cluster of Excellence GreenRobust Heidelberg University 69120 Heidelberg Germany

**Keywords:** *Arabidopsis thaliana*, cambium stem cells, cell division plane orientation, cortical division zone, preprophase band, spindle orientation

## Abstract

- The control of cell division plane orientation is fundamental for organizing developmental processes and shaping bodies of multicellular organisms. In plants, radial organ growth is mediated by the cambium, a stem cell niche embedded in expanding organs and continuously producing xylem and phloem in a strictly bidirectional manner. Cambium stem cells (CSCs) are unique in comparison with other plant stem cells as they consistently divide along their longest axis, clearly overriding the commonly found ‘short axis’ rule. Here, we investigated how cell division plane orientation is controlled in *Arabidopsis thaliana* (Arabidopsis) CSCs.
- We characterized successive microtubule organization during CSC divisions using immunolabelling and histologically compared wild‐type, preprophase band (PPB)‐deficient, and cortical division zone (CDZ)‐deficient mutants.
- We found that division plane orientation is established independently from spindle orientation and the PPB. Instead, the orientation of division planes depends on the CDZ and CDZ‐related PHRAGMOPLAST ORIENTING KINESIN (POK) proteins.
- Robust CSC division orientation is independent of the PPB, challenging the view that the PPB is universally required to stabilize plant cell divisions. Our results highlight the importance of wood‐forming CSCs and their subcellular characterization as an instructive example for the determination of cell division orientation in plants.

## Graphical abstract

Characterization of periclinal and anticlinal cell divisions in dividing cambium stem cells (CSCs). (a) Scheme indicating periclinal and anticlinal cell divisions in CSCs in the Arabidopsis hypocotyl. The orange dashed ring in the middle panel represents the cambium area. The orange cuboids represent individual CSCs. (b–f) Phragmoplast microtubule organization in CSCs undergoing periclinal divisions. (g–k) Phragmoplast microtubule organization in CSCs undergoing anticlinal divisions. Phragmoplast microtubule arrays were detected by immunolabeling in CSCs using an antibody against α‐tubulin (green). Nuclei were stained by DAPI (blue). Cell boundaries are indicated by the white outline. Bars, 3 μm.

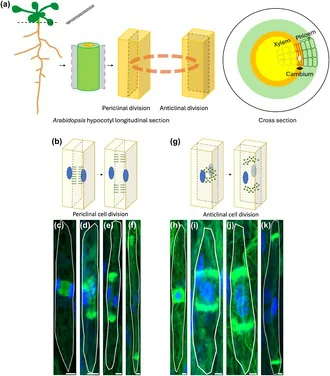

## Introduction

**Fig. 1:**
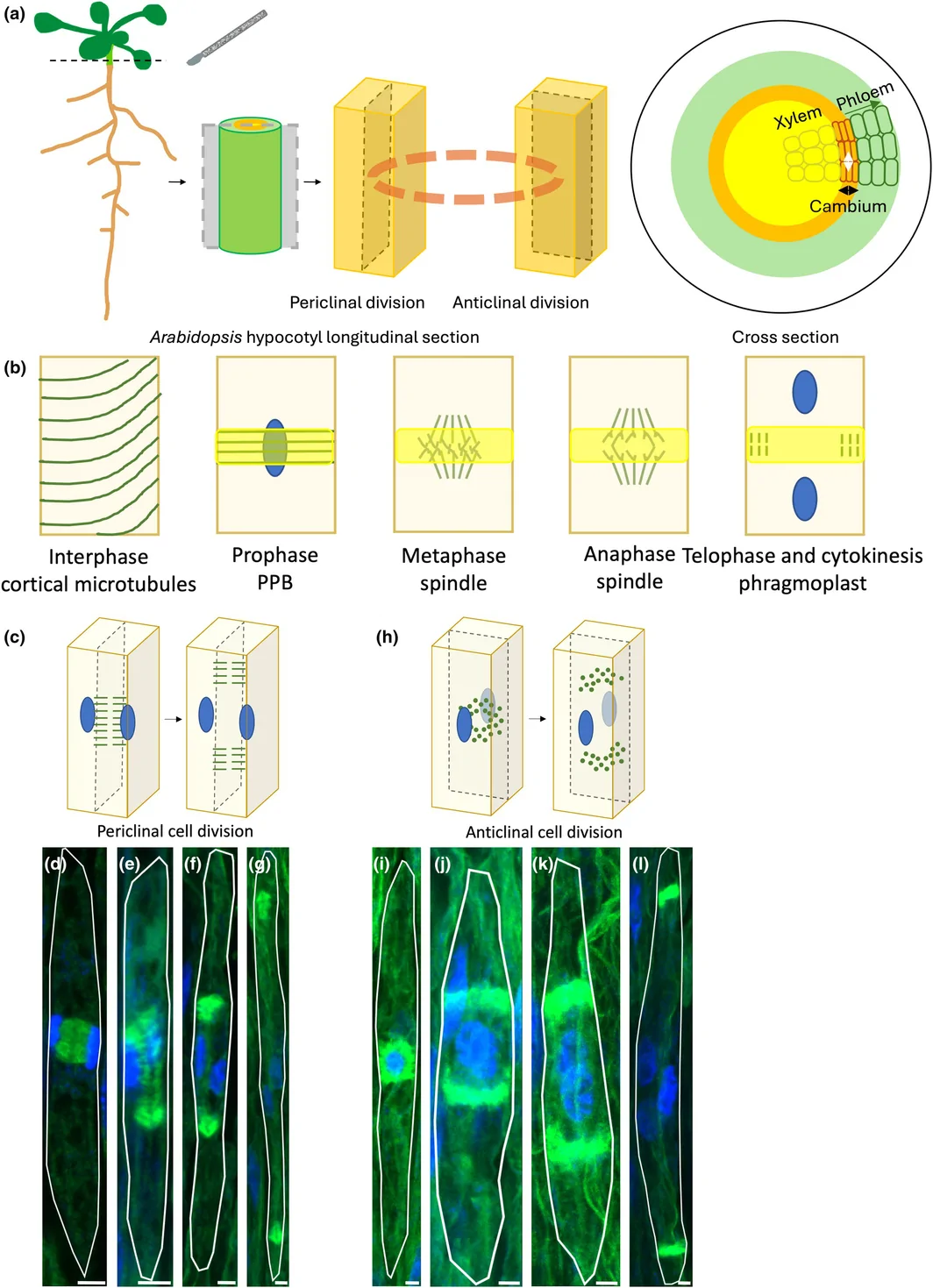
Determination of periclinal and anticlinal cell divisions by visualizing successive stages of phragmoplast microtubule organization after anaphase in dividing cambium stem cells (CSCs). (a) Scheme indicating periclinal and anticlinal cell divisions in CSCs in the Arabidopsis hypocotyl. The orange dashed ring in the middle panel represents the cambium area. The orange cuboids represent individual CSCs. The dashed panels represent longitudinal sections of hypocotyls or the future cell division planes in CSCs. The scheme on the right indicates CSCs (orange), periclinally generating xylem (yellow) or phloem cells (green) and increasing the stem cell domain by anticlinal cell divisions. Black arrows indicate the direction of periclinal cell divisions, white arrows, of anticlinal cell divisions. (b) Scheme indicating diverse microtubule arrays (green) across consecutive cell cycle stages. The preprophase band (PPB) microtubule array specifically marks the cell cortex during prophase, while the cortical division zone (CDZ) was highlighted by the yellow panels. (c–g) Phragmoplast microtubule organization in CSCs undergoing periclinal divisions. (h–l) Phragmoplast microtubule organization in CSCs undergoing anticlinal divisions. Phragmoplast microtubule arrays were detected by immunolabeling in CSCs using an antibody against α‐tubulin (green). Nuclei were stained by DAPI (blue). (d, i) disk phragmoplast, as a dense, solid disk of antiparallel microtubules at the cell center; (e, j) ring phragmoplast, as the microtubules expand centrifugally toward the cell periphery; (f, g, k, l) discontinuous phragmoplast, as microtubules remain where developing cell plates fuse with the plasma membrane. Cell boundaries are indicated by the white outline. *n* = 10 cells. Bars, 3 μm.

Cell division, a fundamental process administering growth and development in multicellular organisms, is precisely regulated in immobile plant cells. Often, plant cells divide along their minimal surface area and generate two equally sized daughter cells by following a ‘shortest axis’ division rule (Errera, 1888). However, divisions decisive for central developmental events frequently deviate from this rule. One example is radial organ growth in dicotyledonous species driven by CSCs, usually bi‐directionally generating vascular tissues, xylem inward and phloem outward (Fischer *et al*., 2019; Shi *et al*., 2019; Smetana *et al*., 2019). CSCs are exceptionally elongated and divide along their longest axis clearly overriding the ‘shortest axis’ rule (Fig. 1a). This property is assumed to be an essential functional trait because CSC derivatives differentiate into interconnected vascular elements whose elongated shape facilitates long distance transport of water, nutrients and organic molecules.

While consistently orienting along the longest cell axis, division planes in CSCs still have typically two possible orientations. Either they orient periclinally, that is, parallel to the organ surface, thereby supporting the increase in the organ radius through the formation of xylem and phloem. Alternatively, they orient anticlinally, that is, perpendicular to the organ surface, thereby increasing the circumferential dimension of the CSC domain (Fig. 1a; Tomescu & Groover, 2019). Regulatory factors influencing the remarkable orientation of these divisions and therefore plant vascular tissue organization are basically unknown. This is partly due to the fact that CSCs are deeply embedded in optically dense tissues, preventing live imaging and direct physical manipulation. Recently, modelling of mechanical stress patterns together with mechanical perturbation in the radially growing Arabidopsis hypocotyl indicated that CSCs experience mechanical stress generated by CSC‐dependent tissue production and used as a stimulus to guide a periclinal orientation of division planes (Höfler *et al*., 2024). Moreover, it was hypothesized that the major stress direction within CSCs aligns with the longitudinal direction of the hypocotyl. This implies that the plane of maximal stress is perpendicular to the plane of minimal area and generally instructs cells to divide along their longest axis. These considerations align well with effects of more traditional mechanical perturbations on isolated cambium domains, during which organized divisions could only be achieved when these domains were under external mechanical pressure (Lintilhac & Vesecky, 1984).

Four specialized microtubule arrays are characterized in plants: the cortical microtubule array, the PPB, the spindle, and the phragmoplast (Hamada, 2014; Fig. 1b). During interphase, microtubules are enriched at the cellular cortex, guiding structures and molecules like extracellular cellulose microfibrils laid down during cell wall synthesis to bear the load of turgor pressure and respond to external mechanical stress (Li *et al*., 2015). At the onset of cell division, during late G2, microtubules organize in PPBs marking the future division plane in the form of a cortical ring. The organization of cortical microtubule arrays, including the PPB during G2/prophase, is accomplished by a protein phosphatase complex (PP2A) targeted to microtubules by TONNEAU1 RECRUITING MOTIF (TRM) proteins (Spinner *et al*., 2013). After the end of prophase, the bipolar spindle apparatus forms perpendicular to the PPB ring during prometaphase (Ambrose & Cyr, 2008), administering the accurate separation of the duplicated nuclear chromosomes in anaphase. Next, the phragmoplast forms between establishing daughter nuclei, directing vesicles to the plane of cell division. There, vesicles fuse and expand the cell plate toward the cortical site previously defined by the PPB (Pickett‐Heaps & Northcote, 1966).

The PPB is part of the so‐called cortical division zone (CDZ), a polarized region corresponding to the selected cell division plane (Smertenko *et al*., 2017; Livanos & Muller, 2019). The PPB is known to facilitate the arrival of CDZ components (Smertenko *et al*., 2017; Livanos & Muller, 2019; Livanos *et al*., 2025). However, there is evidence that another function of the PPB is to limit spindle rotations in order to increase the robustness of cell division orientations. This conclusion is based on the observation that in *trm6;trm7;trm8* (*trm678*) triple mutants compromised in PPB formation, division planes are more variable (Schaefer *et al*., 2017). Nevertheless, PHRAGMOPLAST ORIENTING KINESIN1 (POK1) and POK2, two members of the kinesin‐12 family in Arabidopsis, which co‐localize with the PPB during prophase and function redundantly in maintaining the CDZ (Muller *et al*., 2006; Lipka *et al*., 2014), are still present in *trm678* cytokinetic cells (Schaefer *et al*., 2017; Livanos *et al*., 2025). Underlining POKs' function, *pok1;pok2* double mutants show strongly disoriented cell division planes and a severely dwarfed phenotype (Muller *et al*., 2006), whereas *trm678* mutant phenotypes are considerably milder.

In this study, we characterized CSC divisions on the subcellular level and investigated how CSC's division plane orientation is realized during mitosis. Through systematically determining phragmoplast orientation, we found that *c*. 60% of CSC divisions promote vascular tissue generation by periclinal cell divisions. During metaphase and anaphase, spindle arrays were considerably more variable than in other dividing cells (Schaefer *et al*., 2017), raising uncertainty about the role of PPBs during CSC divisions (Ambrose & Cyr, 2008). This uncertainty was further supported by observations that CSCs are devoid of PPB‐related molecular makers and do not show altered division patterns in PPB‐deficient mutants. By contrast, CSC division plane orientation strongly depended on CDZ components. Our results propose a model in which the robust orientation of CSC divisions along their longest axis depends on directional signals directly acting on CDZ orientation.

## Materials and Methods

### Plant material and plant growth conditions

In this study, the Columbia‐0 (Col‐0) accession of *Arabidopsis thaliana* (L.) Heynh. was used. *trm678* mutants, *pok1;pok2* mutants, and *pTRM7:TRM7‐3xYFP*, *pVHA‐a3:VHA‐a3‐GFP*, *pPOK1:YFP‐POK1*, and *pWOX4:mVenus‐MBD* reporter lines were described previously (Lipka *et al*., 2014; Schaefer *et al*., 2017; Smertenko *et al*., 2017; Lupanga *et al*., 2020; Höfler *et al*., 2024). Arabidopsis seeds were stratified at 4°C for 2 d in the dark before sowing. Seeds were sterilized with a few drops of 70% EtOH and 0.05% Triton X‐100 by pipetting up and down for 1–2 min, depending on the number of seeds. Then, the liquid was carefully poured out. Two hundred to three hundred microliters of 100% EtOH was added for 1 min, and the liquid was poured out on filter paper under the clean bench. Sterilized seeds were sown on 1/2‐strength Murashige & Skoog (1/2MS) solid medium. Plates were kept under short day conditions for 1 wk. One‐week‐old seedlings were transferred from plates to soil and grown in growth chambers under short day conditions (10 h : 14 h, 22°C : 22°C, light : dark, 65% humidity). Three‐week‐old seedlings were then transferred from short day to long day conditions (16 h : 8 h, 22°C : 22°C, light : dark, 50% humidity).

### Immunolabelling

Immunolabelling of microtubules (α‐Tubulin) was described previously (Zhao *et al*., 2020). During this study, we applied this protocol to visualize microtubule organization in cambium stem cells (CSCs) with some modifications. The cell permeabilization buffer contained 1% Driselase (w/v) and 1% Triton X‐100 (v/v) in microtubule stabilizing buffer (Du *et al*., 2021). Sections were incubated in the permeabilization buffer at 37°C for 20–25 min. For immunolabelling of tubulins, Monoclonal anti‐α‐Tubulin antibody (cat no.: T5168; Sigma‐Aldrich) and Goat anti‐Mouse IgG (H + L) Cross‐Adsorbed Antibody, Alexa Fluor™ 488 (cat no.: A‐11001; Thermo Fischer Scientific, Life Technologies GmbH, Darmstadt, Germany), were used as primary and secondary antibodies, respectively. For co‐immunolabelling of tubulins and GFP‐derived fluorescent proteins, besides the antibodies mentioned above to visualize microtubules, GFP Polyclonal Antibody (cat no.: A‐11122; Thermo Fischer Scientific, Life Technologies GmbH) and Donkey anti‐Rabbit IgG (H + L) Highly Cross‐Adsorbed Secondary Antibody, Alexa Fluor Plus 594 (cat no.: A‐32745; Thermo Fischer Scientific, Life Technologies GmbH), were used. For immunolabelling of tubulin and other proteins using wild‐type, *trm678* mutants, and *pTRM7:TRM7‐3xYFP*, *pPOK1:YFP‐POK1* reporter lines, 37‐ to 39‐d‐old seedlings were used to generate longitudinal sections of hypocotyls.

### Histological analysis

Sample preparation and handling for histological analysis were performed as previously described (Wang *et al*., 2019; Wallner *et al*., 2020). Hypocotyls were collected in embedding cassettes and immersed in 70% EtOH for at least 2 d. Samples were then processed using the LEICA ASP200S tissue processor (Leica Microsystems, Wetzlar, Germany) and embedded in paraffin. The embedded samples were sectioned into 10 μm thick sections using the Leica microtome RM2235 (Leica Microsystems). After putting the section onto slides, they were stained by 0.05% toluidine blue and mounted using Micromount Mounting Media (cat no.: 3801731; Leica, Wetzlar, Germany). The mounted slides were left at room temperature for > 2 d. Finally, slides were scanned using the slide scanner Pannoramic SCAN II (3DHistech, Budapest, Hungary). Images were analyzed using the CaseViewer 2.2 software. For wild‐type and *trm678* mutants, cross sections were performed using 28‐d‐old seedlings, and longitudinal sections were performed using 38‐d‐old seedlings. For wild‐type and *pok1;pok2* mutants, cross sections were performed using 32‐d‐old seedlings which had not bolted yet. Longitudinal sections were performed using 42‐d‐old seedlings after bolting.

### Reporter analysis

Sample preparation and manipulation for reporter analysis were performed as previously described (Shi *et al*., 2019; Höfler *et al*., 2024). Briefly, hypocotyls of 38‐d‐old *pVHA‐a3:VHA‐a3‐GFP* were fixed overnight at 4°C by 4% paraformaldehyde (PFA) and washed three times by phosphate buffered saline. For live‐cell imaging of microtubules and YFP‐POK1 visualization, 37‐d‐old seedlings of *pWOX4:mVenus‐MBD* and *pPOK1:YFP‐POK1* lines were used. Hypocotyls were embedded in 7% low melting agarose and free‐hand longitudinal sections were generated using a razor blade. Sections were then placed in a two‐well glass‐bottom dish (cat no.: 80287; ibidi) and immersed in water, with exposed hypocotyl longitudinal sections facing the microscope objective. For analysis of the TRM7‐3xYFP signal intensity in co‐immunolabelling, the Corrected Total Cell Fluorescence (CTCF) was calculated in Fiji to normalize variations in cell size and local background noise, using the formula: *CTCF* = *Integrated Density* − (*Area* × *Background Mean Fluorescence*). For each cell, the region of interest (ROI) was manually integrated to obtain the *Integrated Density* and *Area*. *Background Mean Fluorescence* was determined by averaging the mean gray values of three representative signal‐free regions adjacent to the target cell. All images were analyzed using the original 8‐bit raw data to ensure linear quantification.

### Quantification of spindle and phragmoplast orientation

To analyze the orientation of spindles and/or the phragmoplast in dividing cambium cells of wild‐type and *trm678* mutants, immunolabelling of microtubules and staining of nuclei by 4′,6‐Diamidino‐2‐phenylindol (DAPI, cat no.: D9542; Sigma‐Aldrich) were used. Image analysis was conducted using Fiji (Schindelin *et al*., 2012). CSCs were identified as cells that were adjacent to auto‐fluorescent xylem vessels and showed a very slim morphology. The classification of periclinal and anticlinal cell divisions was strictly based on the spindle/phragmoplast organization and nucleus topology. CSCs which did not show a clear periclinal or anticlinal division orientation were only found when cells were distorted during sectioning or when the section plane had an unfavorable orientation. These ambiguous cases were excluded from the analysis. Only periclinally dividing CSCs were analyzed in angle quantifications. In these CSCs, spindle angles were defined as the angle of aligned chromosomes relative to the longest CSC axis. 0°/90° represented a spindle whose pole‐to‐pole axis was perpendicular/parallel to the longest axis of cambium cells. Phragmoplast angles were defined as the angle enclosed by aligned metaphase chromosomes and the longest CSC axis. Quantification of spindle angles and phragmoplast angles was performed using the ‘angle tools’ in Fiji.

### Confocal microscopy

A Stellaris 8 confocal microscope and a 20× air objective (HC PL APO CS2, 20×/0.75 DRY) and a 63× water objective (HC PL APO CS2, 63×/1.2 WATER) were used in this study. The 405 nm Diode and the white light laser were used for excitation. Image acquisition was performed using a 1024 × 1024 pixel scanning field, 4× line accumulation, pinhole 1 AU and 400 Hz scanning speed. For (co‐)immunolabelling experiments visualizing microtubules (and other proteins) together with nuclei or chromosomes in dividing CSCs, DAPI, GFP, YFP fusion proteins, and FM4‐64 were excited at 405 nm, 489 nm, 514 nm, and 561 nm, and detected at 425–490 nm, 494–547 nm, 519–586 nm, and 582–655 nm, respectively.

### Single cell transcriptome analysis

For single‐cell transcriptome analysis, a previously published dataset (Zhao *et al*., 2025) deposited at Gene Expression Omnibus under accession code GSE224928 was analyzed using the Seurat library of R (Hao *et al*., 2021). Cells with nCount_RNA between 1201 and 9999 and a mitochondrial genome read fraction of < 20% were kept for further analysis. Clustering and UMAP were generated using default settings of Seurat with the parameters ‘dims = 1:15, resolution =1.2’. Marker genes for each cluster were generated by using the ‘FindAllMarkers’ function of Seurat (only.pos = TRUE, min.pct = 0.25, logfc.threshold = 0.25). Individual genes were displayed via the ‘FeaturePlot’ function. The code used for data analysis is found at the GitHub repository [https://github.com/thomasgreb/Liu‐et‐al_CSC‐divisions].

## Results

### Phragmoplast organization reflects CSC division orientation

To investigate the orientation of division planes in CSCs during cytokinesis, we initially visualized successive stages of phragmoplast organization by immunolabeling microtubules in dividing cells. The phragmoplast is organized as a bipolar array of antiparallel microtubules that aid cell plate synthesis at the plane of cell division (Smertenko *et al*., 2017). Usually, the phragmoplast initially forms a disk of microtubules and expands toward the cortex by microtubule turnover. During expansion, the disk then transforms into a ring that becomes discontinuous upon fusion of the cell plate with the parental cell wall (Smertenko *et al*., 2017). Throughout the different stages, phragmoplast organization and nucleus topology typically reveal the future cell division orientation, which we first used to distinguish periclinal from anticlinal CSC divisions (Fig. 1a). As periclinal cell divisions, we categorized those cases in which two nuclei were consistently observed in the same optical plane at both sides of the phragmoplast in radial orientation (Supporting Information Video S1). Disk or ring phragmoplasts appeared in these cases as continuous bipolar structures, separated by a dark line corresponding to the developing cell plate, localized between the two nuclei (Fig. 1c–e). Discontinuous phragmoplasts had expanded away from the cell center and were found close to both the apical and basal cell boundaries (Fig. 1f,g). In contrast to periclinally dividing cells, CSCs undergoing anticlinal cell divisions were characterized by two nuclei present in different optical planes (Video S2). In these cases, the disk phragmoplast had a circular shape, whereas later, phragmoplasts were visible as complete or discontinuous rings (Fig. 1h–l). In total, we found that *c*. 60% of CSC divisions were in periclinal, while *c*. 40% were in anticlinal orientation, among 407 dividing cells analyzed (Table 1). These results indicated that, as proposed before (Spicer & Groover, 2010), CSCs predominantly promote the formation of vascular tissues by conducting periclinal cell divisions, and to a lower extent divide anticlinally increasing the stem cell pool in circumferential orientation. The orientation of the disk phragmoplast in periclinally dividing CSCs appeared slightly variable (Fig. S1), which was not possible to be observed in anticlinally dividing cells because, here, the phragmoplast oriented parallel to the optical plane. Besides the disk stage, the orientation of the phragmoplast microtubule arrays in dividing CSCs perfectly indicated the final division orientation along the longest cell axis in periclinal or anticlinal orientation. In general, we concluded that the final division orientation of CSCs is already reflected by the orientation of the phragmoplast during early cytokinesis.

**Table 1:** Quantification of the number and percentage of dividing cambium stem cells (CSCs) visualizing three distinct phragmoplast microtubule arrays and distinguishing between periclinal or anticlinal divisions.

	Periclinal division	Anticlinal division	In total (number/percentage)
Disk phragmoplast (d, i)	58	26	84 / 21%
Ring phragmoplast (e, j)	46	25	71 / 17%
Discontinuous phragmoplast (f, g, k, l)	148	104	252 / 62%
In total (number/percentage)	252 / 62%	155 / 38%	407 / 100%

Letters refer to Fig. 1.

To calculate the relative duration of cell division stages, we further quantified the proportion of CSCs displaying the three distinct phragmoplast microtubule phases (disk, ring, and discontinuous). These analyses showed that the largest fraction of dividing CSCs (62%) carried a discontinuous phragmoplast, representing the process of microtubule progression from the cell center to the apical and basal cross walls. The smallest fraction (17%) of dividing CSCs showed a ring phragmoplast, which represented the beginning of microtubule array expansion from the cell center to the poles, and 21% of dividing cells each showed a disk phragmoplast, respectively (Table 1). Thus, among different phragmoplast stages in CSCs, the progression of the phragmoplast toward the apical and basal cell boundaries appeared to take the longest. In comparison with other dividing plant stem cells, in which the phragmoplast only needs to span a rather short distance from the cell center to the opposite sides of the parental cell, phragmoplast expansion along the longest CSC axis, which typically extends to 50–100 μm, may therefore result in an overall prolonged cytokinesis. The latter conclusion was consistent with the notion that cytokinesis duration depends on the length of the division plane (Gorst *et al*., 1986).

### Spindle orientation is highly flexible in dividing CSCs

**Fig. 2:**
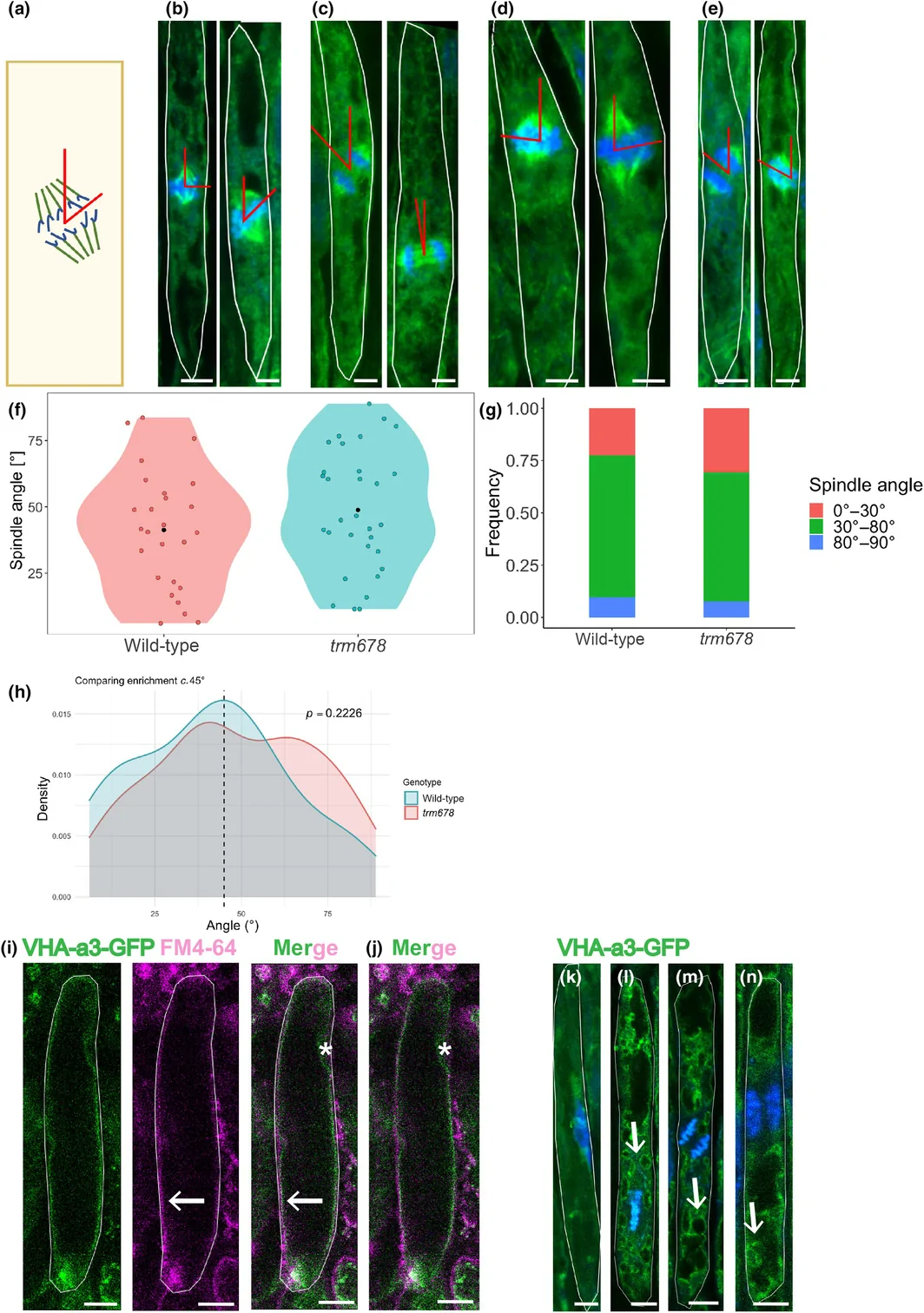
Initial spindle angles in cambium stem cells (CSCs) are flexible in both wild‐type and *trm678* mutants. (a–e) Scheme of spindle orientation quantification in CSCs. Spindle angles (red) were measured by Fiji. Immunolabeling in CSCs was performed using an antibody against α‐tubulin (green). Nuclei were stained by 4′,6‐Diamidino‐2‐phenylindol (DAPI) (blue). Cell boundaries are indicated by the white outline. (b, c) wild‐type; (d, e) *trm678* mutant; (b, d) metaphase spindle; (c, e) anaphase spindle. Bars, 3 μm. (f, g) Quantification of spindle angles in wild‐type (*n* = 26 cells) and *trm678* mutants (*n* = 31 cells) with violin plot (f) or boxplot (g). In (f), the mean is indicated as black dots. Individual data points are shown as pink or green dots. (h) Density distribution of spindle angles in CSCs in wild‐type and *trm678* mutants. The vertical dashed line indicates the 45° reference point. Although the *trm678* mutants appeared more dispersed in the density profile, the overall cumulative distribution showed no statistically significant difference compared with the wild‐type. Statistical analysis by two‐sample KS test. (i, j) Morphology of the vacuole in nondividing CSCs was detected by live‐cell imaging. The arrow indicates the overlap of vacuole (green) and the plasma membrane‐derived signals (magenta) with the exception of the nucleus (asterisk). Cell boundaries are indicated by the white outline (i) or not indicated for full signal visualization (j). *n* = 9 cells. Bars, 10 μm. (k–n) In nondividing (k) and dividing CSCs (l–n), the morphology of the vacuole was detected after paraformaldehyde (PFA) fixation. Nuclei or DNA were stained by 4′,6‐Diamidino‐2‐phenylindol (DAPI) (blue). Vacuole vesicles (green, VHA‐a3) in metaphase (l), anaphase (m) or cytokinesis (n) are indicated by arrows. *n* = 3–5 cells. Bars, 5 μm. A *pVHA‐a3:VHA‐a3‐GFP* line was used in (i–n). Cell boundaries are indicated by the white outline.

Because the orientation of cell divisions along the longitudinal cell axis appeared already set during cytokinesis, we asked whether the same holds true during mitosis. In animal cells, spindle orientation is predominantly regulated by centrosomes and known to be dynamic (Bergstralh *et al*., 2017). In comparison, plant cells lack centrosomes and, determined by the PPB before mitosis, spindle orientation in dividing plant cells is usually very robust (Ambrose & Cyr, 2008). To reveal whether division orientation is already fixed in CSCs during mitosis, we determined spindle orientation during meta‐ and anaphase of cells undergoing periclinal divisions (Video S3). CSCs undergoing anticlinal divisions were excluded from the analysis as the orientation of the spindle perpendicular to the optical plane prevented determining their angles (Video S4). Angles of spindle/chromosome orientation in periclinally dividing cells were measured in relation to the longitudinal CSC axis, with 0° indicating that the orientation of chromosome arrays was parallel to their longest axis (Fig. 2a–c). Interestingly, these measurements revealed a broad variation of orientations covering a range from 0° to 90°, suggesting that the final cell division orientation along the longest cell axis is independent from the orientation of the spindle. A weak maximum of orientations was observed *c*. 45°, as visualized by the density distribution of spindle angles, possibly due to a slight bias of this orientation caused by space constraints in the elongated CSCs (Fig. 2f,h). In comparison with the disk phragmoplast whose orientation rotated within 20° in late division stages, the spindle orientation was therefore considerably more variable.

Of note, the vacuole occupied most of the space in nondividing CSCs, but changed significantly in organization, volume, and size during divisions. Vacuolar distribution is essential for asymmetric cell division of the Arabidopsis zygote and is discussed to influence division plane orientation in other cell types (Panteris *et al*., 2004; Kimata *et al*., 2019). Visualizing vacuole morphology by the *pVHA‐a3:VHA‐a3‐GREEN FLUORESCENT PROTEIN* (*GFP*) marker (Lupanga *et al*., 2020) and the plasma membrane by FM4‐64 staining in live‐cell imaging revealed one central vacuole whose boundaries almost completely overlapped with the plasma membrane‐derived FM4‐64 signal in nondividing cells (Fig. 2i,j). For investigating vacuole morphology in dividing CSCs, we co‐visualized vacuoles and chromosomes after PFA fixation again using the *pVHA‐a3:VHA‐a3‐GFP* marker line. Instead of displaying an intact structure (Fig. 2k), the vacuole had fragmented into small vesicles during metaphase, anaphase, or cytokinesis (Fig. 2l–n; Video S5). We speculate that this compartmentalization of the vacuole during mitosis is a necessary rearrangement that creates sufficient space for microtubule reorganization, chromosome alignment, and segregation in dividing CSCs. In addition, it may allow for the observed variability of spindle orientation.

### Regular cell divisions in cambium stem cells do not rely on PPBs

In light of a rather narrow variation of spindle angles between 0° and 30° during mitosis in root tip cells (Schaefer *et al*., 2017) and of the strict orientation of the final cambium stem cell (CSC) divisions along their longest axis, the broad range of spindle angle variation in dividing CSCs between 0° and 90° was surprising (Fig. 2f). In this context, it is important to note that the PPB is viewed as a stabilizing module aligning to predefined spatial cues, providing a sturdy cortical reference for the orientation of the spindle and the subsequent cell division (Schaefer *et al*., 2017). This feature of the PPB thus prompted us to investigate the role of the PPB in stabilizing the orientation of CSC divisions.

**Fig. 3:**
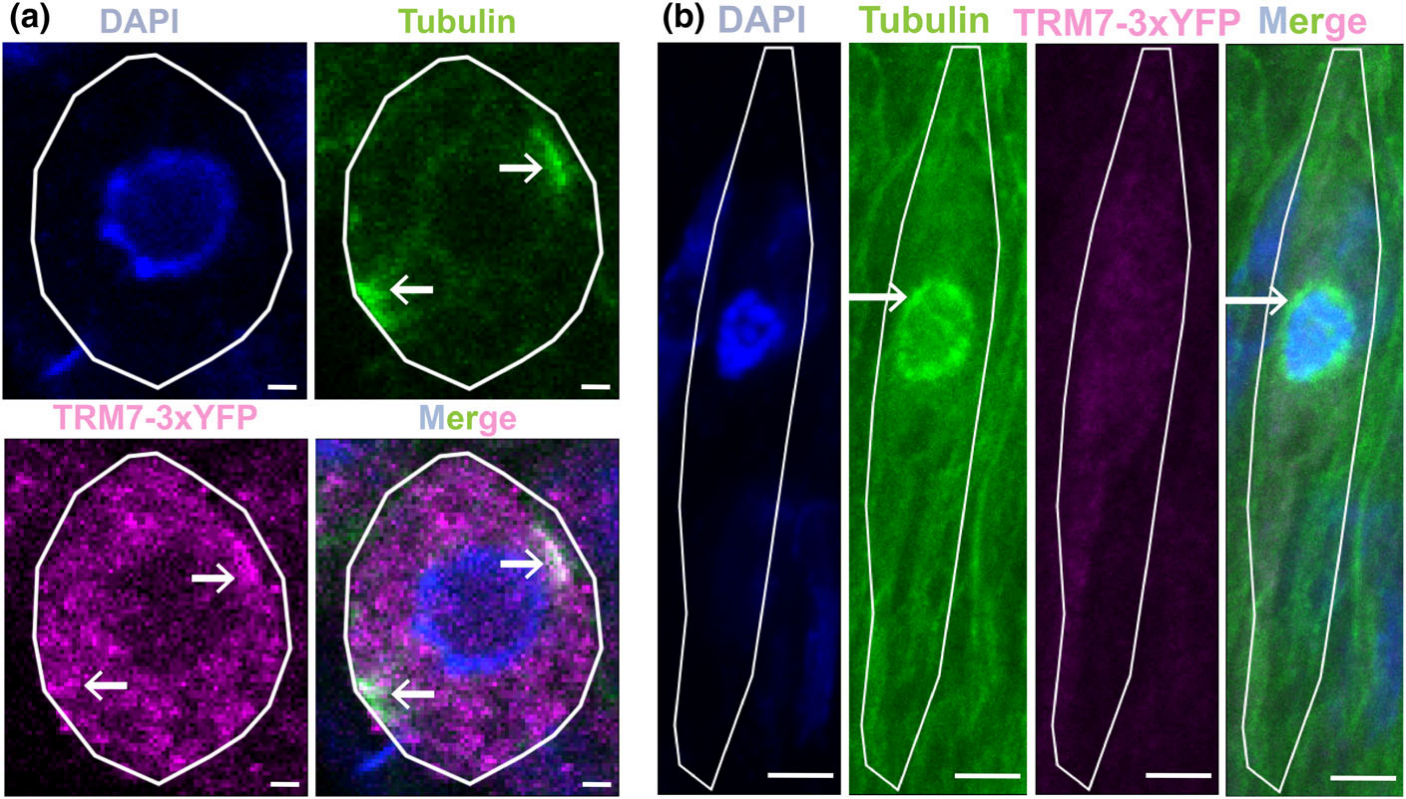
Perinuclear microtubule arrays are detected in dividing cambium stem cells (CSCs). (a) Preprophase band (PPB) microtubule array was detected by co‐immunolabeling in a dividing root tip cell. Arrows point to the PPB microtubule arrays or TRM7‐3xYFP‐derived signals. *n* = 4 cells. Bars, 1 μm. (b) Maximum intensity projections of signals from perinuclear microtubule arrays detected by co‐immunolabeling of tubulin and TRM7 in a dividing CSC using Arrows point to the perinuclear microtubule arrays. *n* = 10 cells. Bars, 3 μm. An antibody against tubulin (green) and an antibody against the yellow fluorescent protein (YFP) protein (magenta). Nuclei were stained by 4′,6‐Diamidino‐2‐phenylindol (DAPI) (blue). Cell boundaries are indicated by the white outline. A *pTRM7:TRM7‐3xYFP* marker line was used in all analyses.

The TRM7 protein serves as a PPB indicator as it colocalizes with PPB microtubules forming a dual, cytoplasmic and cortical ring during late G2 or prophase (Schaefer *et al*., 2017). To visualize TRM7 during cell division we used a TRM7‐3xYELLOW FLUORESCENT PROTEIN (TRM7‐3xYFP) fusion protein expressed under the control of its endogenous promoter (Menges *et al*., 2003; Schaefer *et al*., 2017). Demonstrating the utility of the TRM7‐3xYFP marker, we detected the PPB in dividing root tip cells by co‐immunolabeling microtubules and the YFP‐tagged TRM7 protein. In these cells, TRM7‐3xYFP was localized in both cytoplasmic and cortical areas, where it co‐localized with PPB microtubules (Fig. 3a). However, when conducting co‐immunolabeling of microtubules and TRM7‐3xYFP in CSCs, we found that the TRM7‐3xYFP protein was exclusively present in the cytoplasm and that there was neither a sign of a PPB‐like microtubule formation nor a helical cortical array found in CSCs during interphase (Fig. S2a). By contrast, we observed a high density of perinuclear microtubules identifying these cells as prophase CSCs (Figs 3b, S2b–e; Video S6). Interestingly, this conspicuous microtubule array in dividing CSCs closely resembled the situation of root tip cells in *trm678* mutants in which cells usually do not form a PPB but show a similar assembly of aberrant perinuclear microtubules (Schaefer *et al*., 2017). As already suggested previously, based on ultrastructure analyses (Evert & Deshpande, 1970; Farrar & Evert, 1997; Oribe *et al*., 2001), these observations indicated that CSCs do not form a PPB during their divisions.

To further demonstrate the mitotic specificity of TRM7, we visualized TRM7‐3xYFP expression throughout mitosis in dividing CSCs (Figs S2b–e, S3a–d) and quantified the fluorescence intensity of the TRM7‐3xYFP signal. These analyses showed that the cytoplasmic TRM7‐3xYFP signal was high during prophase, metaphase, and anaphase (characterized by perinuclear microtubule arrays and spindle). By contrast, the TRM7‐3xYFP signal was absent during telophase and cytokinesis, coinciding with the formation of phragmoplast microtubule arrays (Fig. S3e). The results strongly supported a cytoplasmic localization of TRM7‐3xYFP specifically during early mitosis.

### Division orientation in the cork cambium is PPB‐dependent

Radial growth is driven by two concentric bifacial meristems: the vascular cambium and, at the organ periphery, the phellogen. The activity of the phellogen, also known as the cork cambium, generates phellem (cork) outward and phelloderm (cork parenchyma) inward. The phellem–phellogen–phelloderm complex is collectively called the periderm, which forms the outer bark, which is most prominent in woody species (Campilho *et al*., 2020). Given the role of the PPB in predicting cell division plane orientations, we performed histological analyses of hypocotyl cross and longitudinal sections in both wild‐type and PPB‐deficient *trm678* mutants to reveal the functional significance of a PPB absence in CSCs and, as a functional comparison, in the phellogen.

**Fig. 4:**
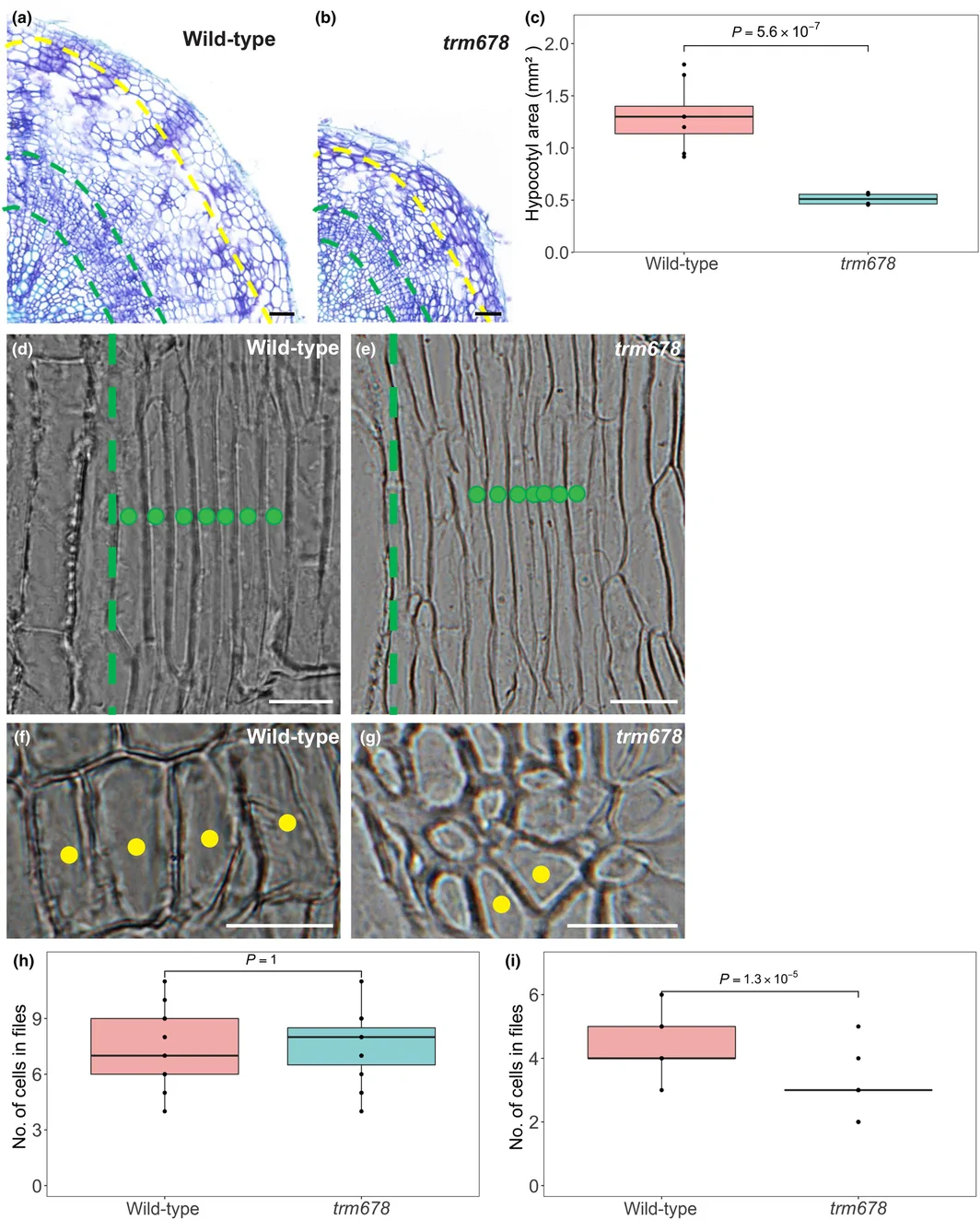
Histological analysis of wild‐type and *trm678* triple mutants. (a, b) Toluidine blue‐stained hypocotyl cross sections from wild‐type (a) and *trm678* mutants (b) embedded in paraffin. The area between two green dashed curves indicates the cambium. The area between the yellow dashed curves and tissue boundary is the periderm. Bars, 50 μm. (c) Quantification of hypocotyl area in wild‐type and *trm678* mutants. The horizontal center lines represent the median, and the upper and lower limits of the box indicate the 75^th^ and 25^th^ percentiles, respectively. The whiskers extend to the maximum and minimum data points from the edge of the box, with data points beyond this range plotted individually as potential outliers. Individual data points are shown as black dots. One‐way ANOVA, *n* = 8 independent seedlings. (d–g) Bright field analysis of longitudinal hypocotyl sections of wild‐type (d, f) and *trm678* mutants (e, g) embedded in Technovit 7100. (d, e) Cambium cell files are seen on the right side of the green dashed lines. The xylem is found on the left side. (f, g) Cell organization in the periderm. Green dots represent CSCs and CSC‐derived cells organized in a file, yellow dots represent phellogen and phellogen‐derived cells organized in a file. *n* = 5–8 independent seedlings. Bars, 20 μm. (h, i) Quantification of the number of cells per file in the cambium (h) and periderm (i) in wild‐type and *trm678* mutants. The horizontal center lines represent the median, and the upper and lower limits of the box indicate the 75^th^ and 25^th^ percentiles, respectively. The whiskers extend to the maximum and minimum data points from the edge of the box, with data points beyond this range plotted individually as potential outliers. Individual data points are shown as black dots. Statistical analysis by One‐way ANOVA, *n* = 5–8 independent seedlings.

When analyzing hypocotyl cross sections, we found the overall hypocotyl area to be decreased in *trm678* compared with wild‐type (Fig. 4a–c), possibly reflecting an overall reduced growth of the *trm678* mutant (Schaefer *et al*., 2017). Importantly, *trm678* plants showed a normal cambium anatomy with highly regular cell division planes, whereas cell arrangement was more irregular in the *trm678* periderm than in wild‐type (Figs 4d–g, S4). Especially, the generation of ordered files of compact cells derived from the phellogen was severely disturbed in *trm678* mutants, unlike the formation of regular cell files in the cambium area (Fig. 4d–i). This observation suggested that, during radial growth, PPB‐deficiency interferes with cell division plane orientation in phellogen stem cells but not in CSCs.

Because previous studies established a role of the PPB in limiting the rotation of the mitotic spindle, we compared spindle orientations between wild‐type and PPB‐compromised *trm678* mutants. CSCs displayed the same variation in spindle rotation during metaphase and anaphase in both wild‐type and *trm678* mutants (Fig. 2a–f). The frequency of CSCs showing spindle angles ranging from 0° to 30°, 30° to 80°, and 80° to 90° was similar between wild‐type and *trm678* mutants, with most spindle angles ranging between 30° and 80° in both wild‐type and *trm678* mutants (Fig. 2g). As mentioned before, such a broad variation of rotation angles was not observed previously in root tip cells of wild‐type plants, which showed variation within 30°, but was similar to the variation of spindle angles in root tips of *trm678* plants (Schaefer *et al*., 2017). Collectively, these results indicated that robust CSC division plane positioning is independent from PPB formation.

### Orientation of CSC divisions depends on CDZ components

**Fig. 5:**
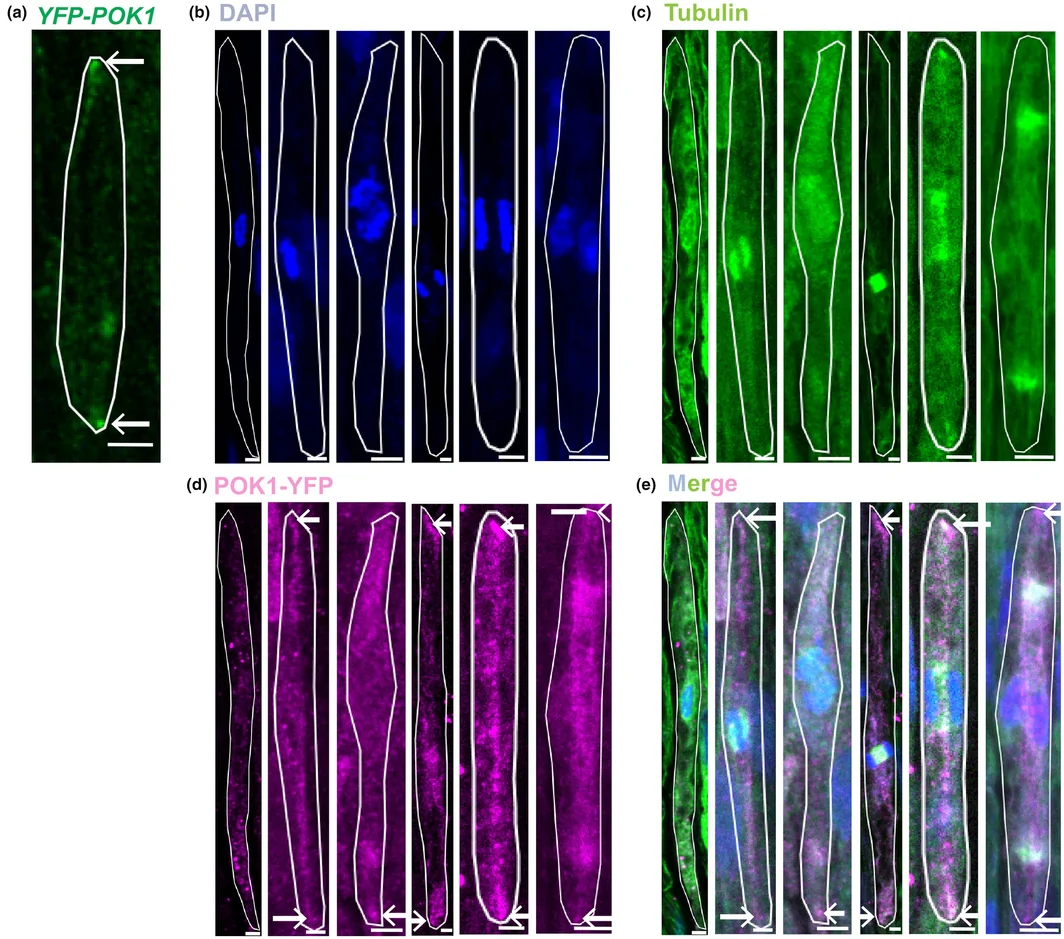
POK1 locates at opposite cambium stem cell (CSC) poles during cytokinesis. (a) POK1 localized specifically at opposite CSC poles in longitudinal hypocotyl sections. *n* = 10 cells. Bar, 10 μm. (b–e) Maximum intensity projections of fluorescent signals during CSC divisions detected by co‐immunolabeling using an antibody against tubulin (green, c) and an antibody against the YFP protein (magenta, d). Nuclei were stained by 4′,6‐Diamidino‐2‐phenylindol (DAPI) (blue, b). (e) Merged images of (b–d). Cell boundaries are indicated by the white outline. From left to right: prophase perinuclear microtubule arrays, metaphase spindle, anaphase spindle, disk phragmoplast, ring phragmoplast and discontinuous phragmoplast. *n* = 2–8 cells. Bars, 3 μm. A *pPOK1:YFP‐POK1* line was used in all analyses. Arrows indicate YFP‐POK1 signals at opposite cell poles. Cell boundaries are indicated by the white outline.

**Fig. 6:**
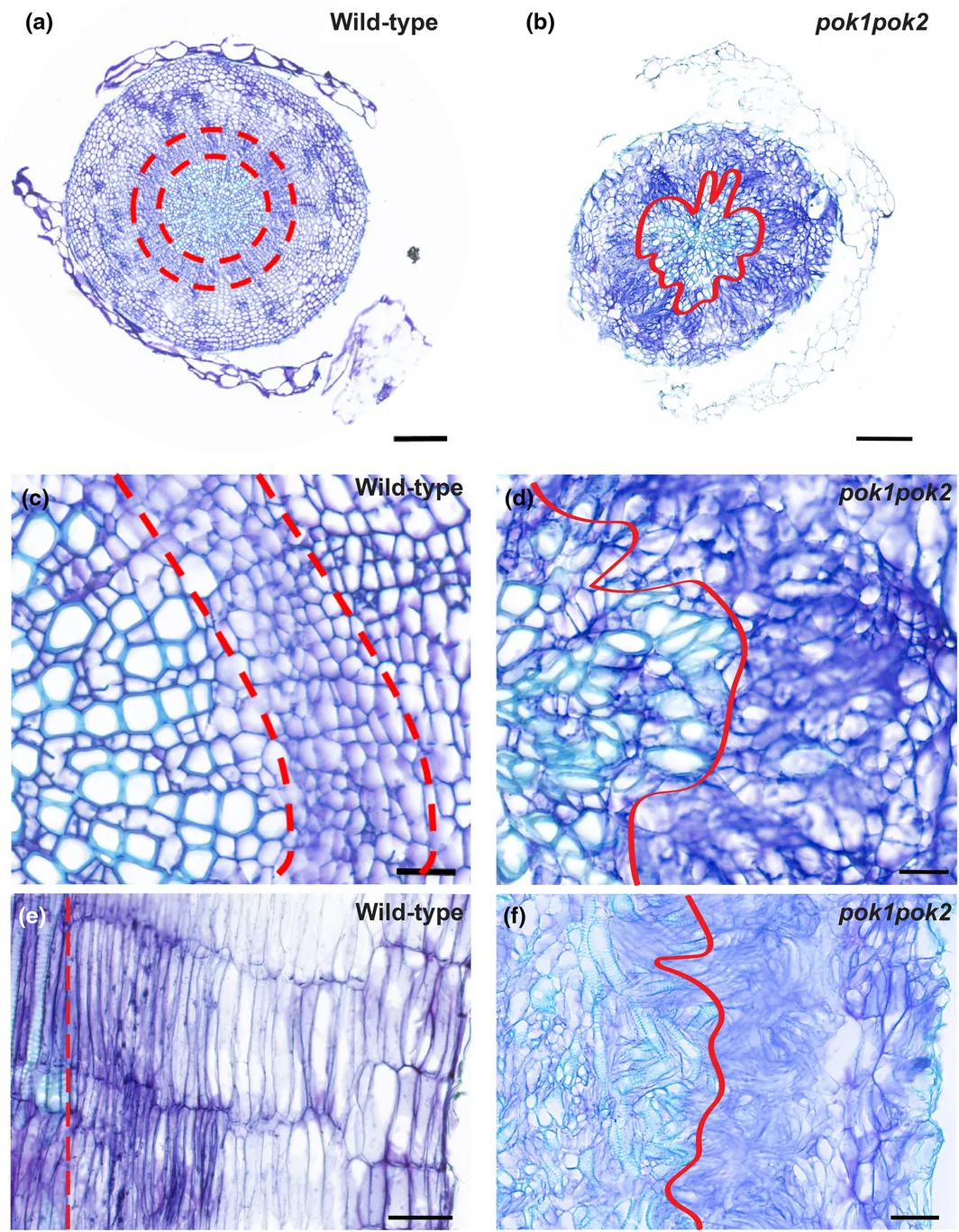
*pok1;pok2* double mutants show highly disorganized vascular tissues. (a, c, e) Hypocotyl cross (a, c) and longitudinal (e) sections of wild‐type stained by toluidine blue. (b, d, f) Hypocotyl cross (b, d) and longitudinal (f) sections of *pok1;pok2* double mutants stained by toluidine blue. The red dashed lines indicate the boundaries of the cambium. The continuous red line indicates the boundaries of the xylem in *pok1;pok2* double mutants. (c, d) are magnifications of (a, b). *n* = 5 independent seedlings. Bars (a, b), 100 μm; (c, d) 20 μm; (e, f) 50 μm.

After having found indications that CSC divisions orient independently from the PPB, we investigated the role of the CDZ in determining CSCs' division orientation. As mentioned, POK1 and POK2 proteins are universally required for the guidance of the phragmoplast toward a preformed CDZ to secure proper division plane orientation (Müller *et al*., 2006) and localize to the CDZ throughout cell division (Lipka *et al*., 2014; Herrmann *et al*., 2018). Indeed, when performing live‐cell imaging in a *pPOK1:YFP‐POK1* reporter line expressing POK1 as a fusion protein to YFP (Lipka *et al*., 2014), we found pairs of focused spots of YFP‐POK1 at the apical and basal ends of CSCs (Fig. 5a). To correlate POK1 localization with different stages of CSC divisions, we next performed co‐immunolabeling of tubulin and the YFP‐POK1 fusion protein using the same *pPOK1:YFP‐POK1* line. These analyses revealed that POK1 was absent from the CDZ during prophase. Upon the formation of the metaphase spindle, POK1 localization at the CDZ became clearly established, appearing as characteristic signals positioned at opposite poles at the cell periphery. This localization remained distinct and stable until the end of mitosis. In CSCs undergoing cytokinesis and showing, among other features, a discontinuous phragmoplast microtubule array between two nuclei, we observed a characteristic POK1 localization at opposing cell ends in images of median cell planes, as part of its ring‐shaped assembly at the CDZ (Fig. 5b–e, Lipka *et al*., 2014). These observations suggested that the CDZ is formed and establishes the position of division planes in dividing CSCs without forming a PPB. Consistently, *pok1;pok2* double mutants showed a dramatically disorganized cambium patterning where the typical concentric cambium organization had disappeared (Fig. 6). In fact, in *pok1;pok2* plants it was not possible to distinguish CSCs from phloem cells, and cell morphologies were substantially altered (Fig. 6). Anatomical alterations of the vascular tissues were already present in young hypocotyls with increasing severity during subsequent developmental stages (Fig. S5). Based on these observations, we concluded that *POK1* and *POK2* genes are necessary for cell division plane positioning and for instructing tissue organization during radial plant growth.

In line with a differential regulation of CDZ vs PPB components, when analyzing single cell transcriptome data of the Arabidopsis hypocotyl published previously (Zhao *et al*., 2025), we found that the CDZ components POK1 and POK2 are expressed in CSCs and, especially, in dividing hypocotyl cells (Fig. S6). By contrast, genes encoding the protein phosphatase scaffolding subunit PP2A1 and TRM8, both involved in PPB formation, showed a rather unspecific activity pattern throughout hypocotyl tissues (Fig. S6). This demonstrated that, in the hypocotyl, regulation of PPB assembly is uncoupled from CDZ formation on the transcriptional level.

## Discussion

Oriented cell divisions generate cell diversity and shape tissues in multicellular organisms. Compared with plant cells, animal cells lack cell walls to maintain their cell shape during growth and development and experience dramatic changes in morphology during mitosis. Typically, dividing animal cells form spheres and remodel the actomyosin cortex for normal bipolar spindle formation (Stewart *et al*., 2011; Lancaster *et al*., 2013). Importantly, the position and orientation of the mitotic spindle determine the orientation of the cleavage furrow and, thus, contribute to the relative size and arrangement of daughter cells (Rappaport & Rappaport, 1974). In comparison, the PPB and the CDZ predict and preserve division plane orientation in plant cells, while spindle orientation usually does not have a strong effect on the placement of the new cell plate (Livanos & Muller, 2019).

The cambium is essential for radial plant growth and executes highly ordered divisions in CSCs. Unlike other plant stem cells, CSCs show a considerable degree of cellular differentiation and divide exclusively along their longest axis, deviating substantially from the ‘shortest axis’ division rule. Considering their unique morphology, we propose that it is instructive to characterize mitosis in CSCs to address the question of how cell division planes are determined in general, a question which has basically not been fully answered in plants. However, CSCs are embedded deeply in optically dense tissues, bringing them out of reach for being observed in intact organs and making them only accessible visually after transverse or longitudinal sectioning. This makes it exceptionally difficult to reveal the dynamics of their subcellular structures during mitosis.

Consistent with previous reports, that CSCs do not form PPBs (Evert & Deshpande, 1970; Farrar & Evert, 1997; Oribe *et al*., 2001), in this study, we found that the PPB reporter protein *TRM7‐3xYFP* did not localize to polarized cortical regions of the cell and we did not find indications for a PPB‐like microtubule assembly in dividing CSCs. Instead, CSCs showed an enriched perinuclear microtubule array as being found in dividing root cells of PPB‐deficient *trm678* mutants. Moreover, *trm678* mutants did neither show alterations in cambium anatomy nor in CSC's spindle orientation. Taken together, these observations indicate that, in spite of their highly ordered cell divisions, there is no relevant function of the PPB in CSCs. In addition to CSC divisions, endosperm cellularization and meiocyte development are examples of processes in which cells undergo well‐ordered, but PPB‐independent, divisions (Otegui & Staehelin, 2004; Brown & Lemmon, 2007). Furthermore, it is important to note that in most nonvascular plants, the PPB is absent or incomplete, or it is present in prophase only in certain cell types (Pickett‐Heaps *et al*., 1999). From an evolutionary viewpoint, the PPB is thus a derived cytoskeletal structure that probably arose with the increased tissue complexity of vascular plants (Rasmussen *et al*., 2013).

What is the functional relevance of an apparent PPB‐deficiency in dividing CSCs? One possibility is that the absence of a PPB is a way to conserve metabolic energy and to accelerate mitosis by avoiding the switch of cortical microtubules from transverse to longitudinal arrays in the very elongated CSCs. Considering its role in stabilizing spindle pole‐to‐pole axis perpendicular to the future division plane, the formation of a PPB in CSCs may also be unfavorable in light of possible spatial constraints. To test this hypothesis, it would be interesting to induce PPB formation in CSCs, for instance by ectopic expression of TRM7 and other potentially absent components of the microtubule organizing TTP complex (Spinner *et al*., 2013). In plant cells conducting PPB‐independent cell divisions, such as gametophytic cells in nonvascular moss gametophore, a diffuse microtubule organizing center (MTOC) was detected during prophase. This acentrosomal microtubule cloud is dispersedly arranged and plays a crucial role in determining spindle and division plane orientation in cells dividing without prior PPB formation (Kosetsu *et al*., 2017). Also, in microspores and endosperm, radial microtubule arrays define division planes in the absence of PPBs (Krupnova *et al*., 2013). However, we did not detect an MTOC‐like or any other specialized microtubule structure during CSC prophase, with the exception of the perinuclear microtubule array reminiscent of *trm678* mutants in root meristem cells. Since the antibody used for immunolabelling only detects α‐tubulin, further experiments to investigate the organization of γ‐tubulin could be conducted to confirm our observations. The molecular mechanisms underlying the variability in spindle orientation during metaphase and factors ensuring the robustness of CDZ component positioning remain unclear, but possibly involve the integration of mechanical cues in dividing CSCs. Further investigations of mechanisms underlying the control of division plane orientation, including the mode of POK recruitment in CSCs, will be essential to fully understand how tissue organization and radial growth are coordinated in the cambium.

## Competing interests

None declared.

## Author contributions

XL and TG designed the study. XL performed experiments and analyzed data. LSS analyzed data. SM provided essential material and conceptual considerations and PL provided technical expertise. XL and TG wrote the manuscript with input from all authors.

## Disclaimer

The New Phytologist Foundation remains neutral with regard to jurisdictional claims in maps and in any institutional affiliations.

## Supporting information


**Fig. S1** Disk phragmoplast is mobile in CSCs.
**Fig. S2** Interphase cortical microtubules and PPB microtubule arrays were detected in CSCs.
**Fig. S3** TRM7‐3xYFP‐derived signals were detected in CSCs undergoing the metaphase and anaphase, but diminished to background levels during the telophase and cytokinesis.
**Fig. S4** Toluidine blue‐stained hypocotyl longitudinal sections from wild‐type and *trm678* mutants embedded in paraffin.
**Fig. S5** *pok1;pok2* double mutants show highly disorganized vascular tissues during radial growth.
**Fig. S6** Analysis of CDZ‐related gene expression in single cell transcriptome data described in Zhao *et al*. (2025).

**Video S1** Maximum intensity projection of a discontinuous phragmoplast microtubule array in a CSC conducting a periclinal division.

**Video S2** Maximum intensity projection of a ring phragmoplast microtubule array in a CSC conducting an anticlinal division.

**Video S3** Maximum intensity projection of a spindle microtubule array in a CSC conducting a periclinal division.

**Video S4** Maximum intensity projection of a spindle microtubule array in a CSC conducting an anticlinal division.

**Video S5** Vacuole morphology in dividing or nondividing CSCs after PFA fixation.

**Video S6** Maximum intensity projection of a perinuclear microtubule array in a prophase CSC.
Please note: Wiley is not responsible for the content or functionality of any Supporting Information supplied by the authors. Any queries (other than missing material) should be directed to the *New Phytologist* Central Office.

## Data Availability

The data that support the findings of this study are available in the supplementary material of this article (Figs S1–S6; Videos [Link], [Link]). The code used for single cell transcriptome analysis is found at the GitHub repository [https://github.com/thomasgreb/Liu‐et‐al_CSC‐divisions].
