# Supplementary material for: The preprophase band is dispensable for robust cell division orientation in wood‐forming cambium stem cells
Source: New Phytol. 2026 Jul 20;252(1):178–91. doi: 10.1111/nph.71449 (PMC13539979; doi:10.1111/nph.71449)
Supplement: Supplementary file 1 [file NPH-252-178-s002.pdf]

New Phytologist

***New Phytologist* Supporting Information**

Article title: The preprophase band is dispensable for robust cell division orientation in wood-forming cambium stem cells

Authors:
Xiaomin Liu, Pantelis Livanos, Laura Sophie Schütz, Sabine Müller, Thomas Greb

Article acceptance date: 1 July 2026

The following Supporting Information is available for this article:

Fig. S1 Disk phragmoplast is mobile in CSCs.

Fig. S2 Interphase cortical microtubules and PPB microtubule arrays were detected in CSCs.

Fig. S3 TRM7-3xYFP-derived signals were detected in CSCs undergoing the metaphase and anaphase but diminished to background levels during the telophase and cytokinesis.

Fig. S4 Toluidine bule-stained hypocotyl longitudinal sections from wild type and *trm678* mutants embedded in paraffin.

Fig. S5 *pok1;pok2* double mutants show highly disorganized vascular tissues during radial growth.

Fig. S6 Analysis of CDZ-related gene expression in single cell transcriptome data described in Zhao et al., 2025.

Video S1 Maximum intensity projection of a discontinuous phragmoplast microtubule array in a CSC conducting a periclinal division.

Video S2 Maximum intensity projection of a ring phragmoplast microtubule array in a CSC conducting an anticlinal division.

Video S3 Maximum intensity projection of a spindle microtubule array in a CSC conducting a periclinal division.

Video S4 Maximum intensity projection of a spindle microtubule array in a CSC conducting an anticlinal division.

Video S5 Vacuole morphology in dividing or non-dividing CSCs after PFA fixation.

Video S6 Maximum intensity projection of a perinuclear microtubule array in a prophase CSC.

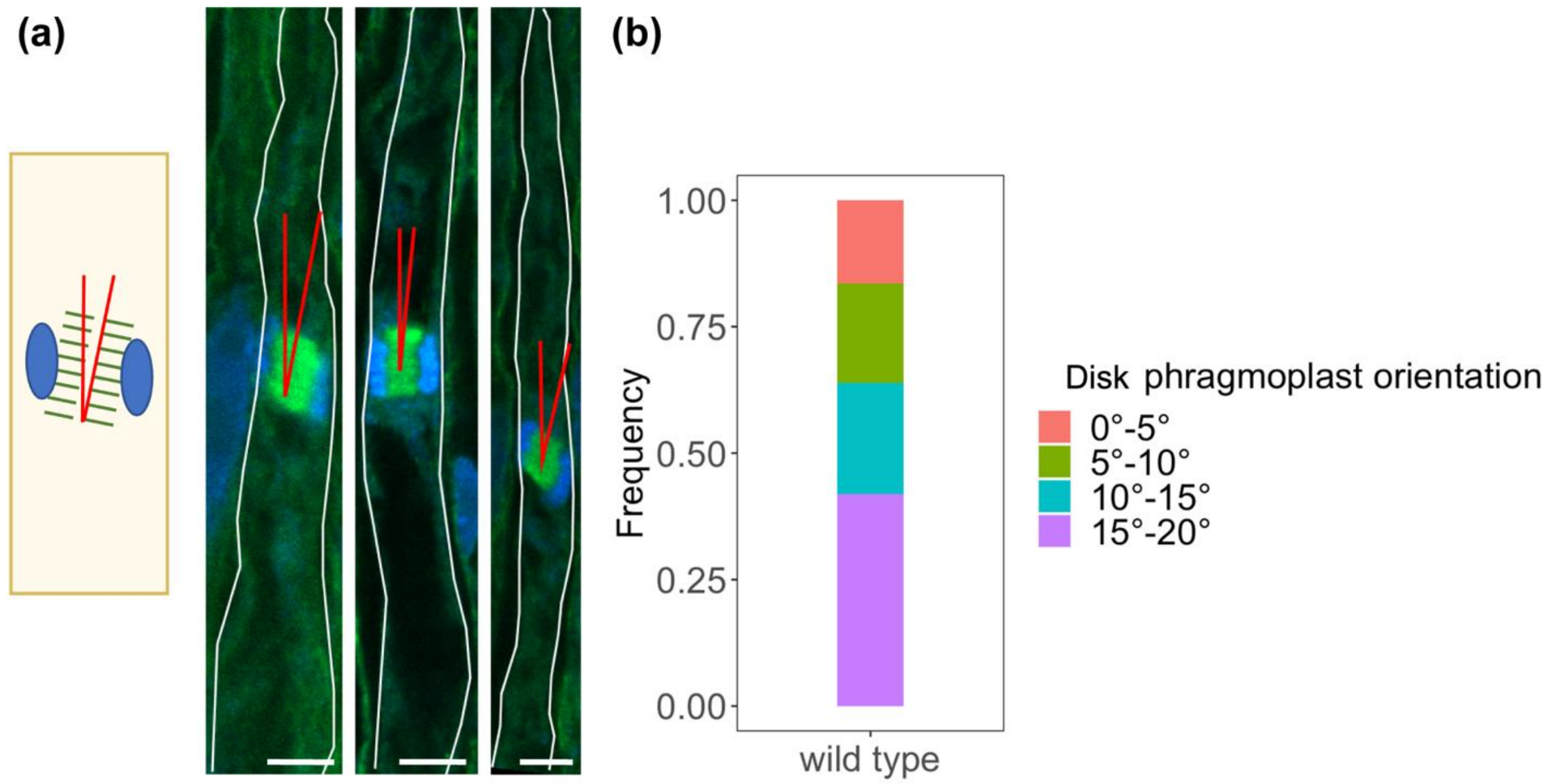

Fig. S1 Disk phragmoplast is mobile in CSCs. (a) Strategy to quantify disk phragmoplast orientation (red angles) in CSCs. Immunolabelling was performed using an antibody against tubulin (green). Nuclei were stained by DAPI (blue). Scale bars = 5 µm. **(b)** Quantification of disk phragmoplast orientation in wild type (n = 25).

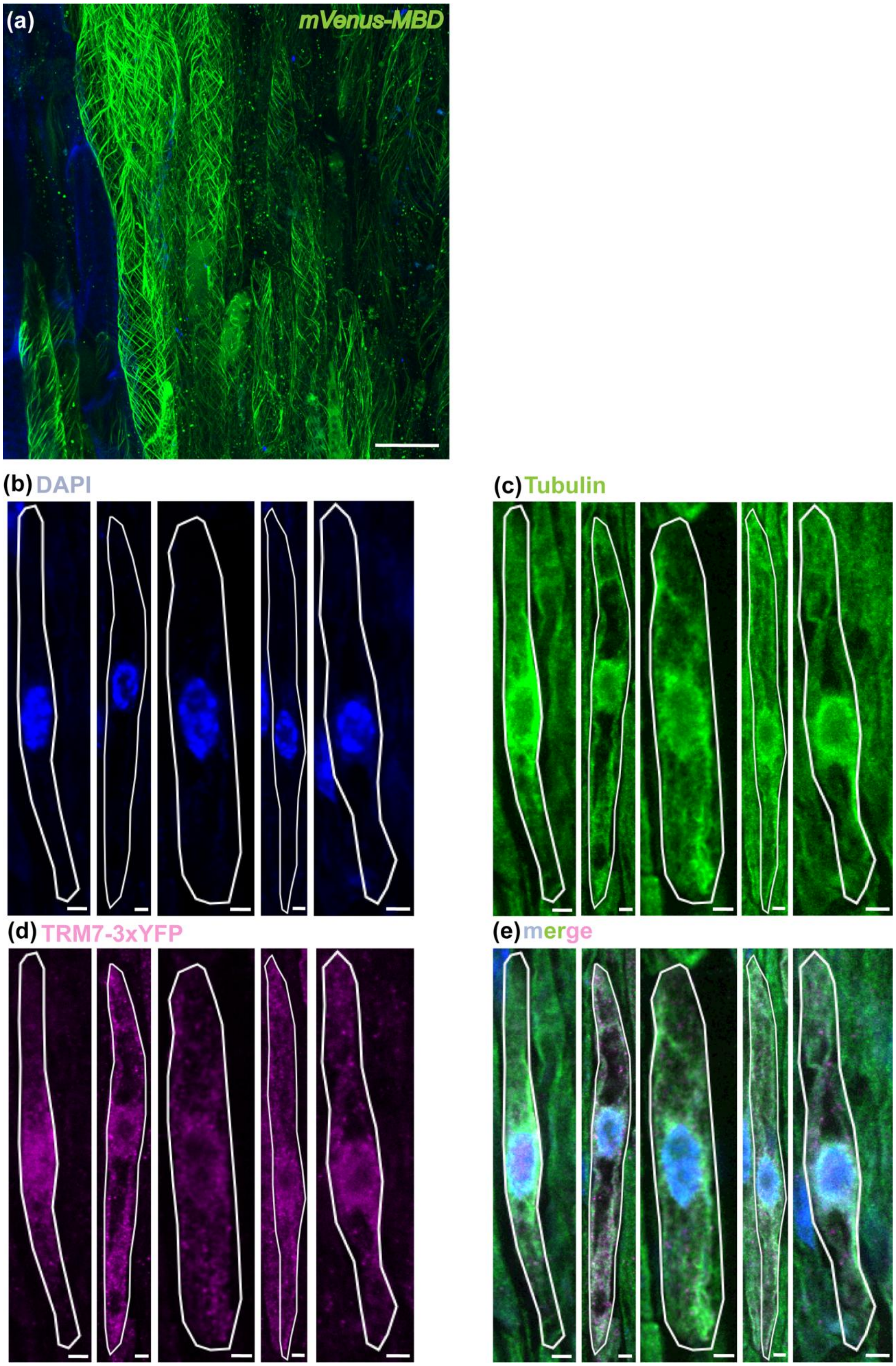
(a)
mVenus-MBD
(b) DAPI
(c) Tubulin
(d) TRM7-3xYFP
(e) merge

Fig. S2 Interphase cortical microtubules and PPB microtubule arrays were detected in CSCs. (a) Maximum intensity projections of signals from interphase cortical microtubules in CSCs visualized by live-cell imaging using the CSC-specific microtubule reporter *pWOX4:mVenus-MBD* (green). Xylem autofluorescence was captured in blue. Scale bar = 20 µm. **(b-e)** Maximum intensity projections of fluorescent signals during five examples of CSCs in prophase generated by co-immunolabeling using an antibody against tubulin (green, c) and an antibody against the YFP protein (magenta, d) using the *pTRM7:TRM7-3xYFP* reporter line. Nuclei were stained by DAPI (blue, b). (e) Merged images of (b-d). Five individual CSCs in prophase are displayed in four separate channels. Cell boundaries are indicated by the white outlines. n = 10 cells. Scale bars = 3 µm.

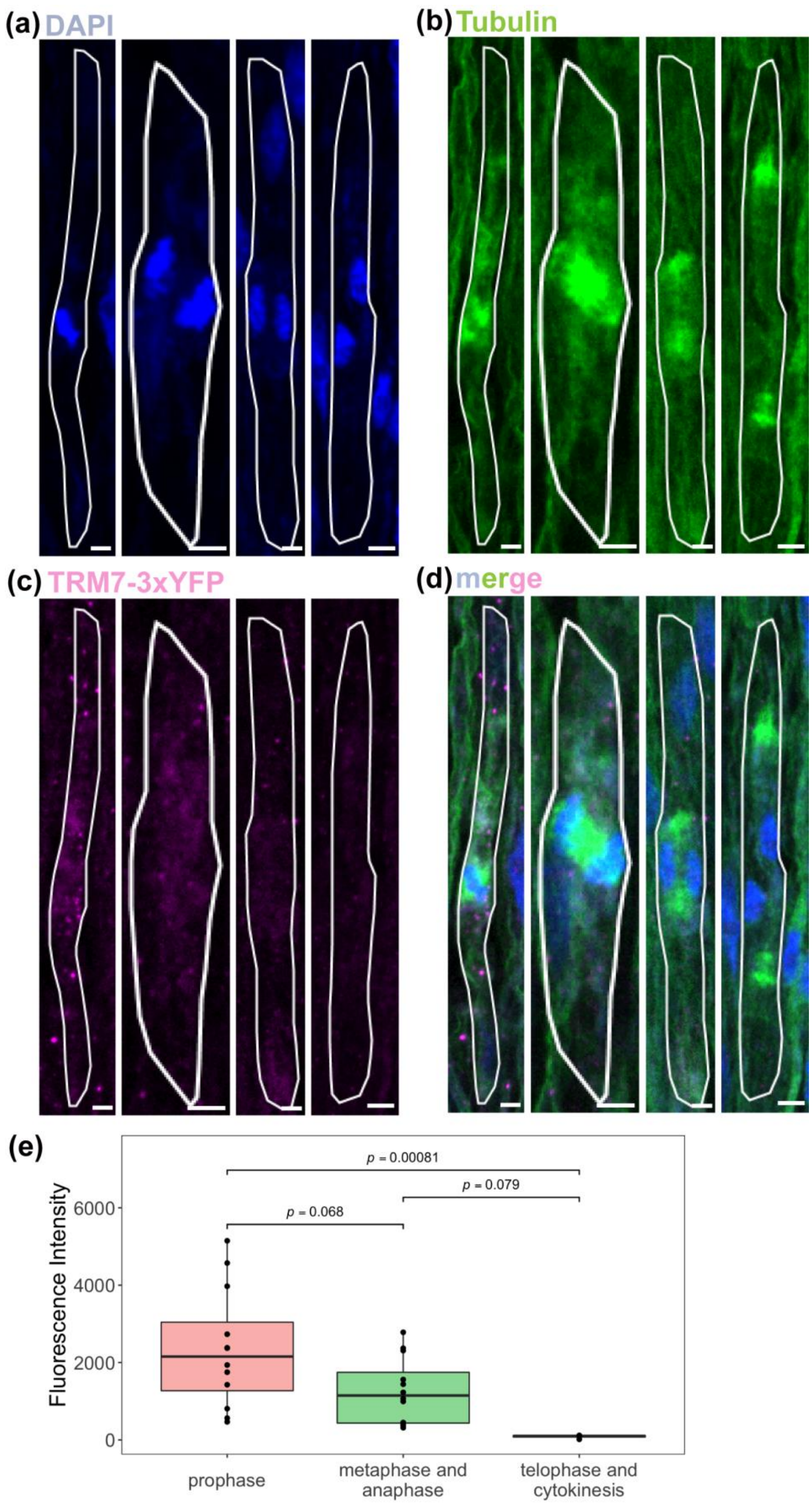
(a) DAPI
(b) Tubulin
(c) TRM7-3xYFP
(d) merge
(e)
Fluorescence Intensity
0
2000
4000
6000
p = 0.00081
p = 0.079
p = 0.068
prophase
metaphase and anaphase
telophase and cytokinesis

Fig. S3 TRM7-3xYFP localization in CSCs during metaphase, anaphase, telophase, and cytokinesis. (a-d) Maximum intensity projections of fluorescent signals in CSCs during metaphase, anaphase, telophase and cytokinesis (from left to right). Cells were stained by DAPI (blue, a), analyzed by co-immunolabeling using an antibody against tubulin (green, b) and an antibody against the YFP protein (magenta, c) using the *pTRM7:TRM7-3xYFP* marker line. (d) shows merged images of (a-c). Cell boundaries are indicated by the white outline. n = 6-10 cells. Scale bars = 3 µm. **(e)** Quantification of the fluorescence intensity of TRM7-3xYFP in CSCs during mitosis and cytokinesis. Values for prophase are extracted from images like shown in Fig. S2. Individual data points are shown as black dots. The horizontal center lines represent the median, and the upper and lower limits of the box indicate the 75th and 25th percentiles, respectively. The whiskers extend to the maximum and minimum data points from the edge of the box, with data points beyond this range plotted individually as potential outliers. Statistical analysis by One-way ANOVA, n = 6-10 cells.

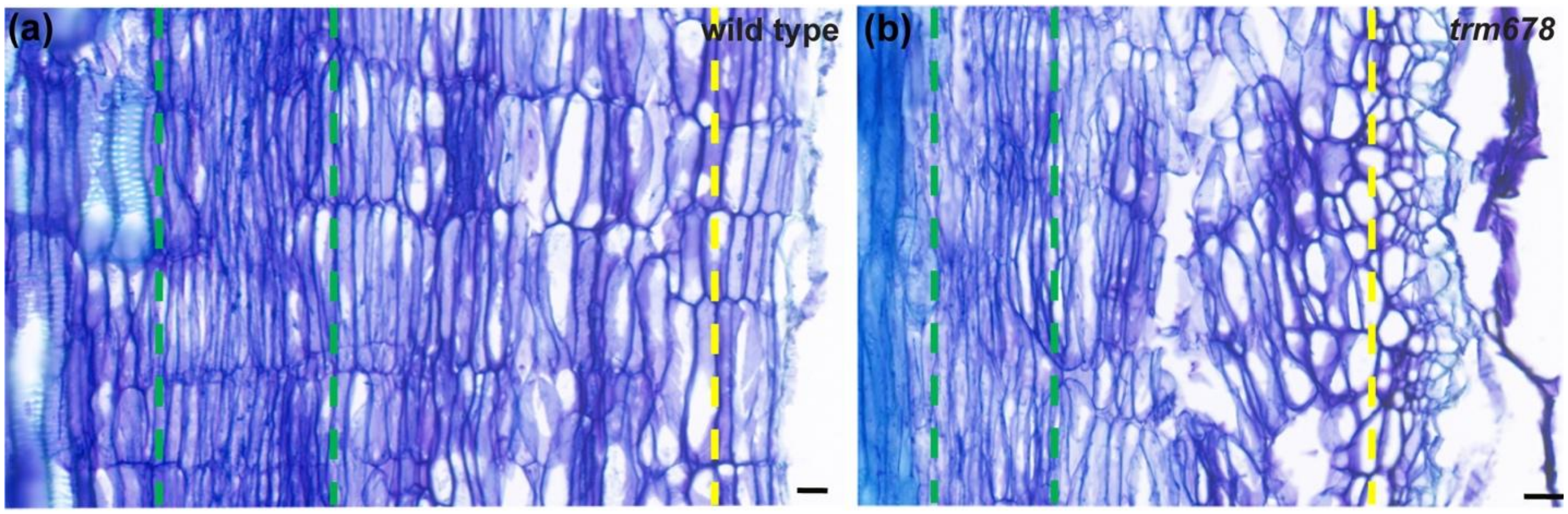

Fig. S4 Toluidine bule-stained hypocotyl longitudinal sections from wild type and *trm678* mutants embedded in paraffin. The area between two green dashed lines is the cambium. The area between the yellow dashed curves and tissue boundary is the periderm. **(a)** wild type, **(b)** *trm678* mutant. Scale bars = 20 µm.

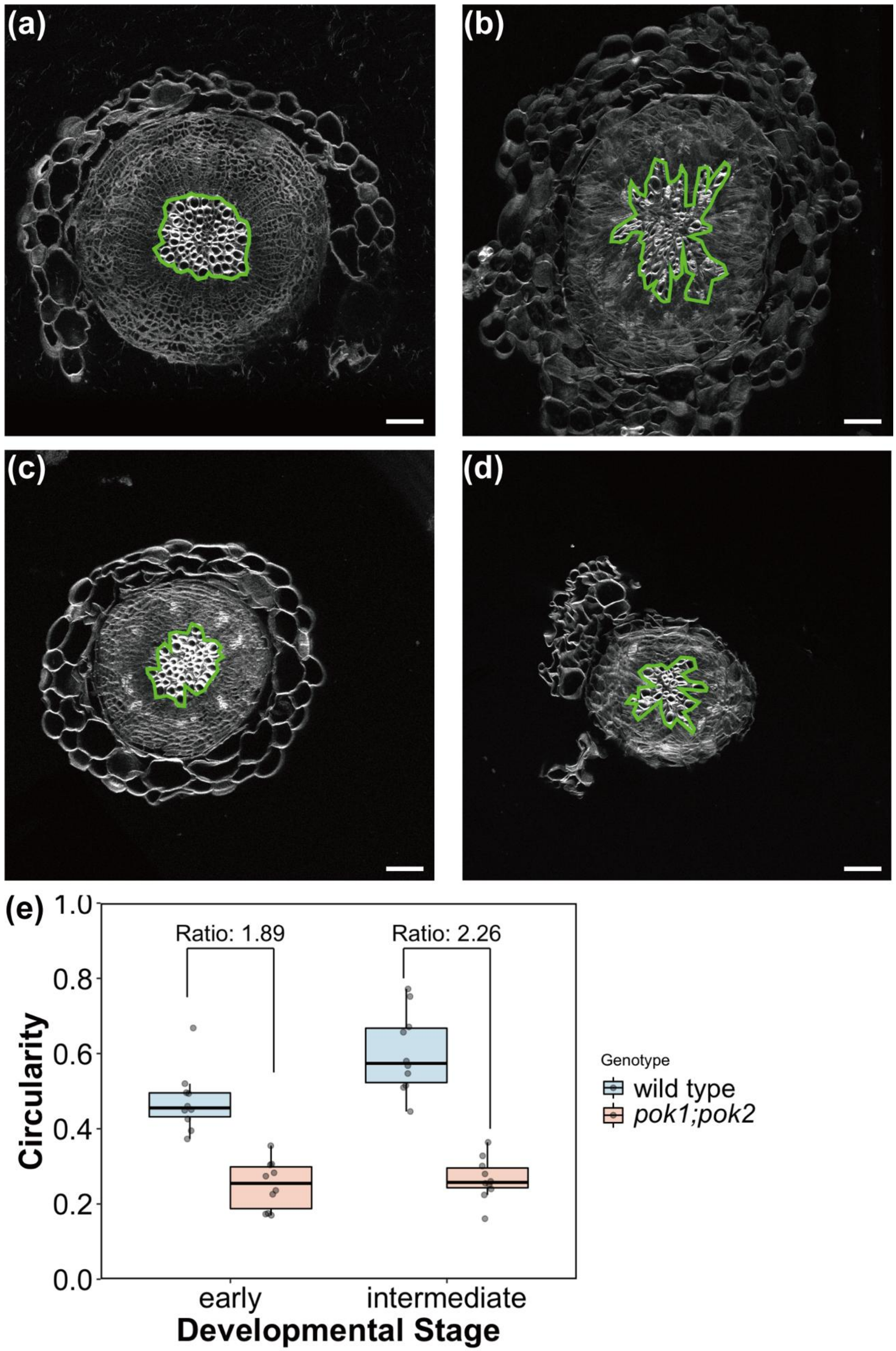
(a)
(b)
(c)
(d)
(e)
Ratio: 1.89
Ratio: 2.26
Circularity
1.0
0.8
0.6
0.4
0.2
0.0
early
intermediate
Developmental Stage
Genotype
wild type
pok1;pok2

Fig. S5 *pok1;pok2* double mutants show highly disorganized vascular tissues during radial growth. (a, c) Hypocotyl cross sections of wild type stained by Direct Red 23. **(b, d)** Hypocotyl cross sections of *pok1;pok2* double mutants stained by Direct Red 23. The green lines indicate the boundaries of the xylem. Scale bars = 50 µm. **(e)** Quantification of the circularity of xylem in wild type and *pok1;pok2* double mutants. The ratio of the average value of xylem circularity in wild type and *pok1;pok2* double mutants during early or intermediate developmental stages were quantified. Individual data points are shown as gray dots. The horizontal center lines represent the median, and the upper and lower limits of the box indicate the 75th and 25th percentiles, respectively. The whiskers extend to the maximum and minimum data points from the edge of the box, with data points beyond this range plotted individually as potential outliers. Black brackets indicate the comparative fold-changes between wild type and *pok1;pok2* double mutants within each developmental stage. n = 10 independent seedlings.

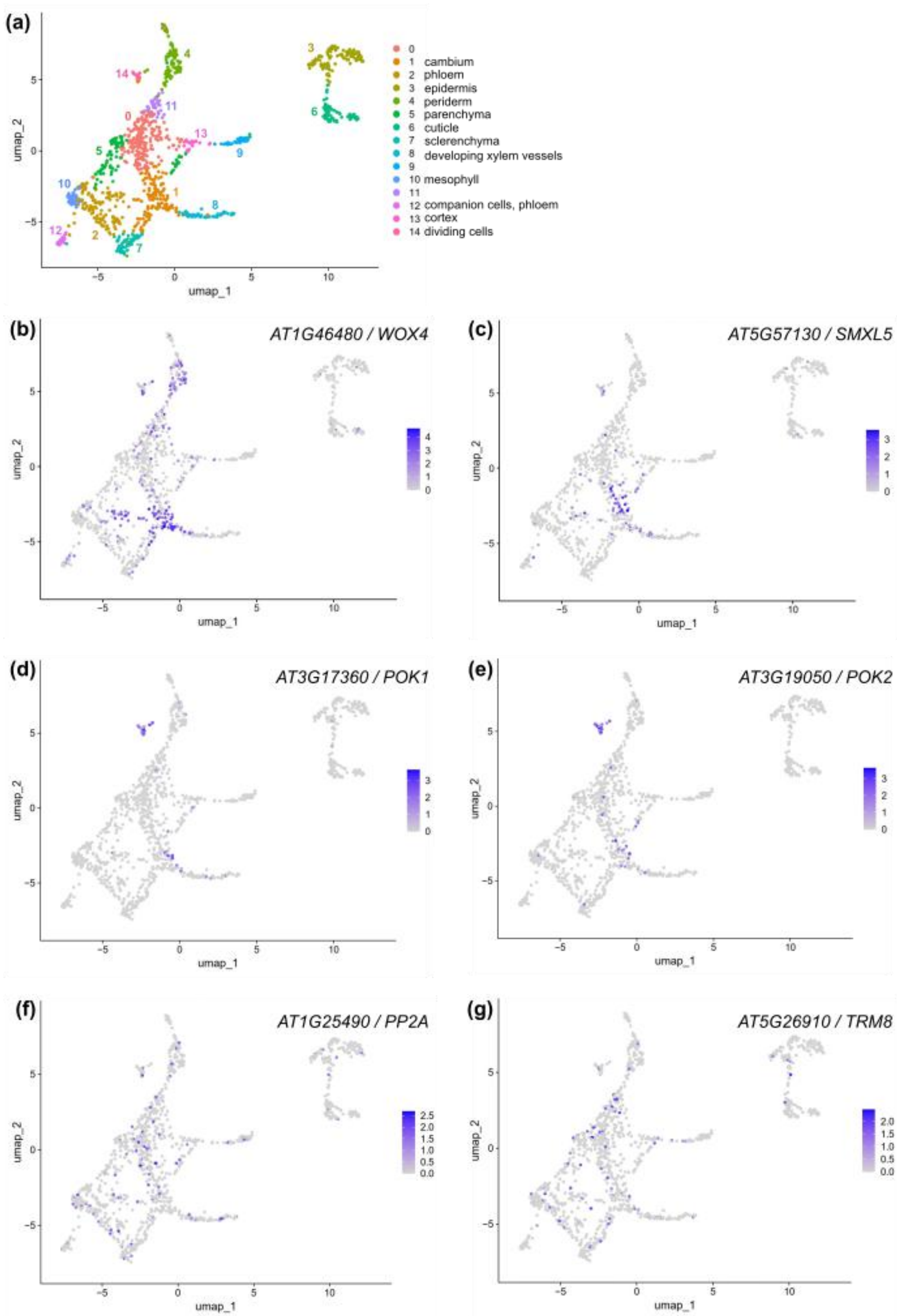
(a)
0
1 cambium
2 phloem
3 epidermis
4 periderm
5 parenchyma
6 cuticle
7 sclerenchyma
8 developing xylem vessels
9
10 mesophyll
11
12 companion cells, phloem
13 cortex
14 dividing cells
umap_1
umap_2
(b) AT1G46480 / WOX4
(c) AT5G57130 / SMXL5
(d) AT3G17360 / POK1
(e) AT3G19050 / POK2
(f) AT1G25490 / PP2A
(g) AT5G26910 / TRM8

Fig. S6 Analysis of CDZ-related gene expression in single cell transcriptome data described in Zhao et al., 2025. (a) UMAP plot of snRNAseq data of 2,061 Arabidopsis hypocotyl nuclei organised in 14 clusters obtained through unsupervised clustering. **(b-g)** UMAP plot of transcript abundance of the two CSC marker genes *WOX4* and *SMXL5* (b, c), the CDZ-related genes *POK1* and *POK2* (d, e), and of the PPB-related genes *PP2A* and *TRM8*. Intensity of blue color indicate relative transcript abundance.

Video Legends

Video S1 Maximum intensity projection of a discontinuous phragmoplast microtubule array in a CSC conducting a periclinal division. Phragmoplast microtubule array was visualized by immunolabeling using an antibody against tubulin (green). Nuclei were stained by DAPI (blue). Slice space = 0.94 µm.

Video S2 Maximum intensity projection of a ring phragmoplast microtubule array in a CSC conducting an anticlinal division. Phragmoplast microtubule array was detected by immunolabeling using an antibody against tubulin (green). Nuclei were stained by DAPI (blue). Slice space = 0.67 µm.

Video S3 Maximum intensity projection of a spindle microtubule array in a CSC conducting a periclinal division. Spindle microtubule array was detected by immunolabeling using an antibody against tubulin (green). Nuclei were stained by DAPI (blue). Slice space = 0.62 µm.

Video S4 Maximum intensity projection of a spindle microtubule array in a CSC conducting an anticlinal division. Spindle microtubule array was detected by immunolabeling using an antibody against tubulin (green). Nuclei were stained by DAPI (blue). Slice space = 0.60 µm.

Video S5 Vacuole morphology in dividing or non-dividing CSCs after PFA fixation. Nuclei or DNA were stained by DAPI (blue). Vacuole morphology was indicated in green. A *pVHA-a3:VHA-a3-GFP* line was used for vacuole visualization.

Video S6 Maximum intensity projection of a perinuclear microtubule array in a prophase CSC. The perinuclear microtubule array was detected by co-immunolabeling in a prophase CSC using an antibody against tubulin (green) and an antibody against the YFP protein (magenta). Nuclei were stained by DAPI (blue). A *pTRM7:TRM7-3xYFP* line was used for the analysis. Slice space = 1 µm.

References

Zhao J, Shi D, Kaeufer K, Song C, Both D, Thier AL, Cao H, Lassen L, Xu X, Hamamura Y, et al. 2025. Strigolactones optimise plant water usage by modulating vessel formation. *Nat Commun* **16**(1): 3854.
